# Diet Plus Inositols, α-Lactalbumin and *Gymnema sylvestre*: The Successful Combo to Restore Body Weight and Metabolic Profile in Obese and Dysmetabolic Patients

**DOI:** 10.3390/nu15143142

**Published:** 2023-07-14

**Authors:** Sabrina Basciani, Maurizio Nordio, Simona Dinicola, Vittorio Unfer, Lucio Gnessi

**Affiliations:** 1 Section of Medical Pathophysiology, Food Science and Endocrinology, Department of Experimental Medicine, Sapienza University of Rome, 00161 Rome, Italy; 2 Department of Experimental Medicine, Sapienza University of Rome, 00161 Rome, Italy; 3 The Experts Group on Inositol in Basic and Clinical Research (EGOI), 00161 Rome, Italy; 4 R&D Department, Lo.Li. Pharma, 00156 Rome, Italy; 5 UniCamillus–Saint Camillus International University of Health Sciences, 00131 Rome, Italy

**Keywords:** myo-inositol, d-chiro-inositol, insulin-sensitizing effect, α-lactalbumin, gut microbiota, intestinal inflammation, *Gymnema sylvestre*, diet, lifestyle

## Abstract

The primary control of dysmetabolic patients is extremely challenging worldwide, with inadequate dietary habits and sporadic physical activity among the key risk factors for metabolic syndrome onset. Nowadays, there is no exclusive treatment for this condition, and considering that preventive measures usually fail, new therapeutic approaches need to be proposed and investigated. This present pilot study compared the effects of diet alone and in association with a combination of myo-inositol and d-chiro-inositol in their 40:1 ratio, α-lactalbumin, and *Gymnema sylvestre* on different metabolic parameters in obese dysmetabolic patients. To this purpose, 37 patients with BMI between 30 and 40 and fasting blood glucose between 100 and 125 mg/dL were divided into two groups: (*i*) the control group followed a hypocaloric Mediterranean diet, (*ii*) while the study group was also supplemented with a daily dosage of two sachets, each one containing 1950 mg myo-inositol, 50 mg d-chiro-inositol, 50 mg α-lactalbumin, and 250 mg *Gymnema Sylvestre*. After a 6-month treatment, all parameters improved in both groups. Nevertheless, the treated group experienced a greater improvement, especially concerning the variation from the baseline of HOMA index, triglycerides, BMI, body weight, and waist circumference. These findings support the supplementation with myo-inositol and d-chiro-inositol in the 40:1 ratio, α-lactalbumin, and *Gymnema sylvestre* as a therapeutical strategy to potentiate the beneficial effects induced via dietary programs in dysmetabolic patients.

## 1. Introduction

Nowadays, the term “metabolic syndrome (MetS)” comprises a cluster of comorbid pathologies, including hypertension, central obesity, insulin resistance, and atherogenic dyslipidemia, which together increase the risk of developing type II diabetes mellitus (T2DM) and cardiovascular diseases (CVD) [1]. MetS is a major and escalating public-health concern as well as a clinical challenge worldwide, especially in Western countries [2,3]. As its prevalence ranges from 10% to 84%, the International Diabetes Federation (IDF) established that one-quarter of the world’s adult population is affected by MetS.

Because of the complex interplay between genetic and environmental factors in the etiopathogenesis of MetS, and since it is frequently underrated especially by patients themselves, its clinical identification and treatment are pivotal efforts to reduce the risks of related diseases.

Management guidelines recommend lifestyle changes above all, primarily body weight loss and exercise, while, in the case of failure in using such approaches, pharmacological intervention is advised to reduce the specific risk factors and achieve a faster result.

In this context, natural compounds, in association with a healthy and balanced diet, could be helpful in improving altered metabolic parameters in patients affected by MetS.

The Inositol family comprises natural molecules involved in several biochemical and metabolic functions in different organs and tissues [4]. Myo-inositol (myo-Ins) is the best-known and the most bioavailable isoform among the nine stereoisomers. It is basically incorporated into eukaryotic cell membranes as phosphatidyl-myo-inositol, serving as a precursor of inositol triphosphate (InsP3), which is the second messenger of several hormones, like follicular stimulating hormone (FSH), thyroid stimulating hormone (TSH), and insulin. Endogenously, myo-Ins is synthetized from glucose-6-phosphate (G6P), which is isomerized to inositol-3-phosphate (InsP3) by D-3-myo-inositol-phosphate synthase enzyme (inositol synthase, Ino1, or MIPS1). Then, using the inositol monophosphatase-1 (IMPA-1 or IMPase) enzyme, InsP3 is dephosphorylated into free myo-Ins. This latter could also be obtained via the dephosphorylation of inositol-1,4,5-trisphosphate (InsP3) and inositol-1,4-bisphosphate (InsP2). The human body, especially the kidney, is capable of synthetizing about 4 g/day of myo-Ins.

Besides the endogenous synthesis, in humans, a large amount of myo-Ins (about 1 g/day) is also provided by dietary intake: vegetables, oats, corn, and cereals are foods that are particularly rich in this polyol.

For more than 20 years, myo-Ins have been considered a safe and effective therapy, especially for the management of Polycystic Ovary Syndrome (PCOS), an endocrine-metabolic disorder characterized by altered insulin signaling [5]. Under insulin stimulation, tissue-specific epimerase enzymes convert myo-Ins into its stereoisomer d-chiro-inositol (d-chiro-Ins) [6]. Myo-Ins and d-chiro-Ins, by promoting glucose uptake ad glycogen synthesis, respectively, may exert their insulin-sensitizing effect, thereby reducing systemic glucose and decreasing insulin requirements, which consequently correlate with lower circulating insulin concentrations [7].

Several scientific evidence demonstrated that myo-Ins and d-chiro-Ins, when combined in a 40:1 ratio, can restore the physiological glucose metabolism and reduce insulin resistance [8] as well as have beneficial effects on cholesterol and triglycerides [9,10]. Recently, a meta-analysis stated that inositol supplementation has a positive effect on body mass index (BMI) reduction, suggesting the use of this molecule as an adjunct therapy to improve anthropometric indices and glycemic response [11].

However, despite the widely accepted beneficial effects of inositols on metabolic, hormonal, and reproductive abnormalities, some patients do not respond to inositol treatment. The cause of this “inositol resistance” is not yet well understood, mostly because the trials do not assess the differences between responders and non-responders in terms of hormonal and metabolic profiles.

In addition, a wealth of studies confirmed that myo-Ins intracellular depletion is a common condition in both diabetic animal models and human subjects [12,13]. Different mechanisms are involved in inositol depletion, mainly the alteration of gut microbiota composition (dysbiosis), which leads to intestinal inflammation and reduced inositol absorption [14].

Observational findings achieved over the past two decades, suggested that the gut microbiota may contribute to the metabolic health of the human host; therefore, a condition of dysbiosis has been linked to the pathogenesis of different metabolic disorders, including insulin resistance, obesity, and T2DM [15]. α-lactalbumin (α-LA) is a milk whey globular protein produced via the epithelial cells of the mammary gland, with prebiotic, mucoprotective, and anti-inflammatory action [16]. Due to its prebiotic activity, α-LA may support the establishment of proper intestinal flora, preventing or recovering gut dysbiosis and related metabolic effects. Such protein also acts as a factor enhancing the intestinal absorption of other micronutrients, such as vitamins and microelements. Indeed, α-LA positively affects inositol transport from the intestinal lumen to the bloodstream, favoring inositol absorption and bioavailability. Preliminary in vitro evidence suggests that peptides deriving from the digestion of α-LA may modulate tight junction permeability, allowing for the increased absorption of both myo-Ins and d-chiro-Ins [17,18]. As a matter of fact, the combination of α-LA and myo-Ins has proved to reduce some PCOS-related alterations better than myo-Ins alone, leading to overcoming the “inositol resistance” phenomenon [19,20,21]. The clinical study by Montanino and colleagues indicated that adding 50 mg of α-LA to 2 g of myo-Ins twice a day, restored ovulation in 86% of inositol-resistant women with PCOS.

Importantly, α-LA dietary supplementation significantly ameliorated serotonin synthesis and release from serotonergic neurons leading to positive effects on mood and anxiety, very often impaired in dysmetabolic and/or obese patients [19].

To date, herbal medicines have gained much popularity to treat the above-mentioned diseases because of their safety and the lack of side effects. Among them, *Gymnema sylvestre* is a medicinal plant belonging to the *Asclepiadaceaea* family. It is a slow-growing, perennial, medicinal woody climber found in central and southern India and tropical Africa with potent anti-obesity and anti-diabetic activities. Its leaves contain gymnemic acids, a mixture of at least 17 different saponins, acidic glycosides, and anthraquinones with antidiabetic activity. In Ayurvedic medicine, *Gymnema sylvestre* is considered one of the most relevant botanicals for the treatment of various diseases, such as cardiovascular diseases, asthma, eye complaints, cancer, inflammation, diabetes, and obesity. In particular, several studies evidenced that this wild herb may restore the correct balance of glucose and insulin in diabetic patients by reducing the intestinal absorption of glucose [22,23].

Besides having marked anti-diabetic properties, *Gymnema sylvestre* presents anti-obesity features [24], as it decreases body weight and BMI [25].

On these premises, the concomitant administration of these above-mentioned natural substances could be prospected as adjuvant therapy in combination with a dietetic regimen.

Hence, the aim of this study was to evaluate the effects of a hypocaloric Mediterranean diet associated with the oral supplementation of myo-Ins and d-chiro-Ins in a 40:1 ratio, α-LA, and *Gymnema sylvestre*, on insulin, glucose, lipid metabolism, and anthropometric measures in dysmetabolic and obese patients, who did not require pharmacological treatments.

## 2. Materials and Method

### 2.1. Study Design and Participants

This was a pilot, open label, controlled, and interventional study conducted from April 2022 to October 2022 at Section of Medical Pathophysiology, Food Science and Endocrinology, Department of Experimental Medicine, Sapienza University of Rome. This study has been carried out in full accordance with Good Clinical Practice guidelines and the Declaration of Helsinki and registered on ClinicalTrials.Gov (Identifier: NCT05348304; Approval number: CE6422; Board name: Kemeso). All patients provided written informed consent.

Men and women aged 25–65 years, with BMI between 30 and 40, and fasting blood glucose between 100 and 125 mg/dL were enrolled. Key exclusion criteria included (*i*) diabetes diagnosis; (*ii*) concomitant use of hypoglycemic drugs or food supplements containing inositols, *Gymnema sylvestre*, and α-LA; (*iii*) intolerance to at least one of the previous components. A total of 37 patients meeting these criteria were divided into two groups. The study group (*n* = 21 patients) was treated with a daily dosage of two sachets, each one containing 1950 mg myo-Ins, 50 mg d-chiro-Ins, 50 mg α-LA, 250 mg *Gymnema sylvestre*, and 7.5 mg zinc (Eudiamet^®^ 40:1, Lo.Li. pharma S.r.l., Rome, Italy) in addition to a hypocaloric Mediterranean diet. The dosage was two sachets a day, one hour before meals (taken 12 h apart), dissolving the content in a glass of water (200 mL).

Regarding the diet, each patient in both groups did not have free access to food. In particular, all of them followed the well-known hypocaloric Mediterranean diet, which consists of fruit, vegetables, olive oil, legumes, cereals, and fish and a fixed percentage of carbohydrates, mostly mono- and polyunsaturated fats and proteins (55, 25, and 20, respectively).

The control group (*n* = 16 patients) was represented by patients that followed only a hypocaloric Mediterranean diet without assuming the supplement.

All the patients were monitored for 6 months.

### 2.2. Study Outcomes

The aim of this study was to evaluate the effects of a hypocaloric Mediterranean diet in association with the supplementation of myo-Ins and d-chiro-Ins in 40:1 ratio, α-LA, and *Gymnema sylvestre*, on glucose and lipid metabolic parameters including insulin, blood glucose levels, Homeostasis Model Assessment (HOMA) index, BMI, weight, triglycerides, high-density lipoprotein (HDL) cholesterol, low-density lipoprotein (LDL) cholesterol, and waist circumference.

### 2.3. Statistical Analysis

Data were analyzed via Mann–Whitney U test (2018 GraphPad Software 8.0.1, La Jolla, CA, USA) performed on the absolute variation (Δ); values are indicated as median [25th percentile–75th percentile]. We considered a *p*-value ≤ 0.05 as statistically significant.

## 3. Results

### 3.1. Baseline Sample Characterization

A total of 37 patients participated in this pilot study: 21 subjects were assigned to the study group (15 females, 6 males) and 16 to the control group (12 females, 4 males). One patient in the study group discontinued this study because of the antidiabetic therapy. Baseline parameters, such as age and anthropometric measures, were similar between the treatment and the control group.

### 3.2. Fasting Insulin, HOMA Index, and Blood Glucose

After 6 months, in both groups, patients registered an improvement in terms of reductions in fasting insulin, HOMA index, and blood glucose. Nevertheless, the treatment group, which received the supplementation of myo-Ins and d-chiro-Ins in a 40:1 ratio, α-LA, and *Gymnema sylvestre*, experienced a larger, albeit not significant, improvement with respect to the control group (Table 1).

In detail, fasting insulin in the treated group decreased more than in the control one. Median changed from 19.70 [16–26.10] to 13.70 mcU/dL [12.10–18.70] in the intervention group while from 15.65 [14.72–24.37] to 14.25 mcU/dL [12.12–16.20] in the control group. The same trend was observed for both the HOMA index and fasting blood glucose.

In the case of the HOMA index the median value varied from 4.90 [3.97–7.20] to 3.45 [3–4.80] in the intervention group and from 4.20 [3.90–6.35] to 3.45 [3.10–4.02] in the control group, while fasting blood glucose median changed from 107 [103–113.50] to 101 mg/dL [97.25–103.75] in the intervention group and from 105.50 [103.75–109.25] to 100.50 mg/dL [95.5–104.50] in the control group.

### 3.3. Lipid Profile

Like the metabolic glucose asset, the lipid profile was also ameliorated in both groups.

As a matter of fact, total, HDL and LDL cholesterol, as well as triglycerides improved in all patients, reaching a statistical significance only as far as the reduction in triglycerides in the treated group is concerned (Table 2).

Total cholesterol changed from 237.50 [229.50–259] to 222 mg/dL [208–241] in the intervention group and from 244.00 [232.75–274.75] to 230.50 mg/dL [211.75–252] in the control group. HDL cholesterol shifted from 39.50 [35.25–43.50] to 49.00 mg/dL [44–51] in the intervention group and from 41 [37–46] to 51 mg/dL [48–54.25] in the control group. LDL cholesterol changed from 156 [145–168.75] to 146 mg/dL [124–163] in the intervention group and from 156 [147.25–176.50] to 140.50 mg/dL [123.75–160.50] in the control group.

Considering the triglycerides, the median changed from 213.50 [193.25–239] to 162 mg/dL [155–172] in the intervention group (*p* < 0.001) and from 226 [208.25–255] to 189.50 mg/dL [174.50–208.75] in the control group (*p* < 0.001). When comparing the values between the two groups after 6 months, we found a significantly higher reduction in the intervention group (*p* < 0.01).

### 3.4. BMI, Weight, and Waist Circumference

In both groups, patients registered an improvement in terms of BMI, weight loss, and reduction in waist circumference.

However, differences reached significance only concerning the reduction in BMI in treated patients with respect to baseline (*p* < 0.001) (Table 3).

In detail, the BMI median in the study group reduced from 31.30 [30.50–33.27] to 29 [28.3–31] and from 34.25 [32.40–37] to 32.75 [30.90–34.52] in controls.

Moreover, weight median changed from 84.75 [79.25–96.77] to 79.20 kg [75.40–89.90] in the treated group while from 90.8 [86.35–100.75] to 88.8 kg [82.37–96.80] in the control group. For waist circumference, the median shifted from 98 [96–110] to 92 cm [90.50–105] in the intervention group and from 103 [95.75–110.50] to 100 cm [90.75–105.50] in the control group.

### 3.5. Delta Variation Analysis

**Figure 1:**
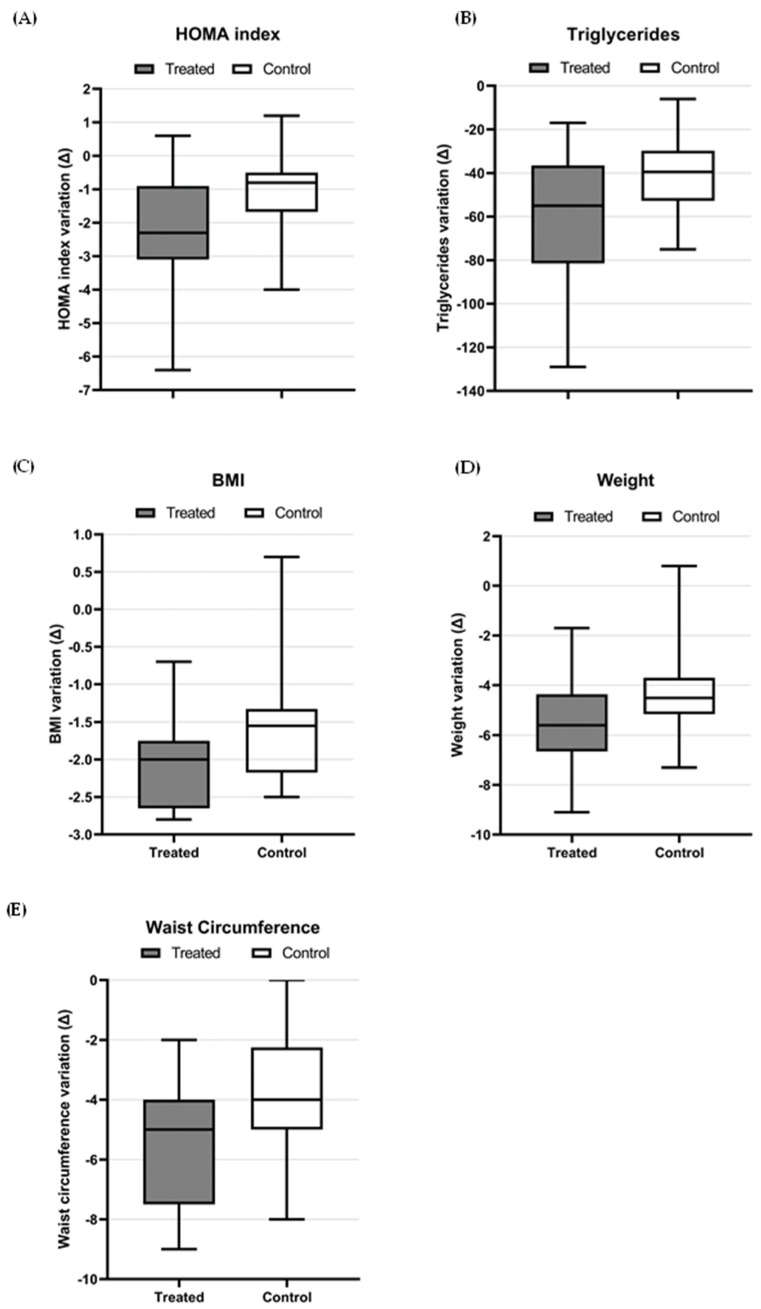
Absolute variation (Δ) of HOMA index (**A**), triglycerides (**B**), BMI (**C**), weight (**D**), and waist circumference (**E**) from baseline to 6 months between treated (hypocaloric Mediterranean diet plus inositols, α-LA, and *Gymnema sylvestre*) and control groups (hypocaloric Mediterranean diet); all Δ were significant with *p*-value < 0.05.

To better understand the advantage of associating myo-Ins and d-chiro-Ins, α-LA, and *Gymnema sylvestre* with a hypocaloric Mediterranean diet with respect to the diet alone, the absolute variation (Δ) from baseline to 6 months was calculated between the two groups. Running this analysis, significant differences were found in five of the considered parameters: HOMA index, triglycerides, weight, BMI, and waist circumference. All these variations are reported in Figure 1.

## 4. Discussion

Metabolic disease constitutes a pathogenetically and clinically heterogeneous entity, which has been highlighted as a major public health emergency during the past 50 years due to its accompanying multimorbidity. Apart from morbidity, there is no doubt that metabolic disease is a major contributor to mortality.

The presence of specific abnormal parameters, such as elevated waist circumference and triglycerides, reduced HDL cholesterol, high blood pressure, and increased fasting plasma glucose, define a diagnosis of MetS.

Despite the prevalence of MetS may depend on the various diagnostic criteria, along with the composition of the studied population (in terms of sex, age, and ethnicity), the percentage of globally affected people is alarmingly high [2]. Although such syndrome is a collection of different cardiometabolic risk factors, its pathophysiology seems to be largely attributable to insulin resistance. Namely, insulin resistance appears as the cornerstone of MetS, formerly often referred to as “insulin resistance syndrome”. As such, a huge part of the patients suffering from MetS is at high risk of developing T2DM [26].

In adipose tissue, insulin resistance impairs the anti-lipolytic activity of insulin, leading to an exacerbated lipolysis that increases circulating free fatty acids (FFAs).

Moreover, there is accumulating evidence on the fact that inflammatory cytokines possibly derived from adipose tissue, such as tumor necrosis factor-α (TNF-α), contribute to the development of insulin resistance. An acute rise of TNF-α in plasma further exacerbates lipolysis, therefore, increasing circulatory FFA levels. Inflammatory cytokines such as TNF-α or interleukine-6 (IL-6) induce insulin resistance via the activation of serine/threonine kinases such as Jun *N*-terminal kinase (JNK), nuclear factor-kappa B (NF-κB), and mammalian target of rapamycin (mTOR) that promote inhibitory serine phosphorylation of IRS-1 (insulin receptor substrate 1). Through the two main mechanisms described above, the accumulated fat, especially visceral adiposity, is thought to be a major contributor to insulin resistance, thus stressing the importance of energy imbalance (i.e., high caloric intake and low energy expenditure) as a pivotal causative factor.

Insulin resistance is also known as a major contributor to dyslipidemia. The increased flux of FFAs to the liver stimulates the production of very low-density lipoproteins (VLDL). Under physiological conditions, insulin inhibits the secretion of VLDL into the systemic circulation, but this action is impaired when the liver becomes insulin resistant. In the setting of insulin resistance, the increased flux of free fatty acids to the liver also increases hepatic triglyceride synthesis, leading to hypertriglyceridemia, a hallmark of insulin resistance.

On these premises, the clinical management of this syndrome is first and foremost based on recommending healthy lifestyle programs intended as a combination of sufficient physical activity and a well-balanced diet [27,28,29,30,31,32,33]. However, very often, adherence to these lifestyle changes is weak and inefficacious, and in certain circumstances, an additional intervention appears to be mandatory.

Despite the limited number of patients, our pilot study demonstrated how obese and slightly hyperglycemic patients improved their metabolic parameters after a 6-month supplementation with myo-Ins and d-chiro-Ins (in their physiological 40:1 ratio), α-LA and *Gymnema sylvestre*, in association with a hypocaloric Mediterranean diet, more than in the case of diet alone.

As a matter of fact, the variation from the baseline of HOMA index, triglycerides, weight, BMI, and waist circumference reached a significant improvement in the treated group compared with the control group, which was only on a hypocaloric diet.

Among the several natural compounds from which patients with MetS can take advantage, inositols, α-LA, and *Gymnema sylvestre* have a prominent role due to their positive effects on glucose metabolism, insulinemia, intestinal inflammation, and lipid profile.

Almost concomitantly, Larner and Nestler were the first authors to propose inositols as chemical insulin mediators, stimulating the interest of the scientific community in the insulin mimetic properties of inositols and the investigation of their usefulness in gynecological and endocrinological clinical practice [34]. Nowadays, inositol treatment is among the most widespread non-pharmacological approach for insulin resistance and all the related pathological morbidities, most of all hyperinsulinemia and PCOS [34].

Indeed, a wealth of papers agree to state the effectiveness and the safety of inositol supplementation, mainly with reference to myo-Ins and d-chiro-Ins in their 40:1 ratio, to normalize insulin resistance and hyperinsulinemia in dysmetabolic patients, like overweight and obese women affected by PCOS, in which systemic and ovarian inositol ratio resulted impaired [5,34,35].

Moreover, some studies reported that dietary myo-Ins deficiency results in lipid accumulation in the liver of rats [36] and fish [37,38].

D-chiro-Ins also plays a pivotal role in lipid metabolism as it regulates steroid production in rats and adipocyte differentiation in human cells [39].

As additional consideration, one should note that inositols may induce the trans-differentiation of white adipose tissue (WAT) to brown adipose tissue (BAT), which is usually altered in the case of obesity. In this view, a recent study demonstrated that human differentiated adipocytes assumed the typical feature of brown adipose tissue after treatments with myo-Ins and d-chiro-Ins. This also correlated with the expression of peroxisome proliferator-activated receptor gamma (PPAR-γ), which is a key target for lipid metabolism that favors the transition from white to brown adipocytes [40].

On the other hand, by binding the taste receptors on the tongue, *Gymnema sylvestre* allows reducing the sweet perception and intensity. Many studies demonstrated that this wild herb significantly reduces fasting blood glucose and glycated hemoglobin in different patients with metabolic disorders [41]. Moreover, when orally administered to rats with hyperlipidemia, *Gymnema sylvestre* reduces total cholesterol, LDL, and triglycerides. [42]. Considering its role in the regulation of carbohydrate and lipid metabolism, it was quite predictable that the combination with inositols would strengthen their positive effects, and, to the best of our knowledge, this is the first study demonstrating this synergy.

Lastly, the presence of α-LA implemented even more the benefits of myo-Ins/d-chiro-Ins and *Gymnema sylvestre*. Such milk protein, when orally administered, can pass unchanged through the stomach, exerting a protective effect against inflammation and insulin resistance, which are typical features of obesity [43]. In the gut, α-LA improves the intestinal absorption of some micronutrients, as well as of inositols, increasing their bioavailability and therapeutical efficacy. Nowadays, the recent literature is focusing on the prebiotic activity of α-LA by suggesting its ability in modulating some beneficial intestinal microbial populations [19]. This is an aspect that should not be underestimated, as, very often, subjects with obesity and/or MetS display gut dysbiosis, intended as an alteration in the composition of intestinal microbiota [44,45].

In this regard, a recent review highlighted that some bacterial strains, including those stimulated via α-LA (*Lactobacillus acidophilus*, *Bifidobacterium short*, *Bifidobacterium longum*, and *Bifidobacterium infantis*), are associated with significant improvements in glycated hemoglobin and HOMA index in patients with T2DM, suggesting the positive effects of α-LA in restoring altered metabolic parameters [19]. On these premises, α-LA can certainly contribute to the establishment of proper intestinal flora, preventing and/or recovering gut dysbiosis and related metabolic effects. Clearly, all these properties make this molecule a good candidate as the missing piece in the treatment of dysmetabolism.

## 5. Conclusions

Overall, this is the first study demonstrating how the combination of inositols (specifically myo-Ins and d-chiro-Ins in the 40:1 ratio), α-LA, and *Gymnema sylvestre* can be beneficial as adjuvant treatment for patients with impaired metabolic profile and alterations in body weight, especially when allied to proper dietary habits.

Our conclusions must acknowledge the small sample size of this pilot study, which may represent a limitation. Thus, additional clinical studies are necessary to corroborate and expand related findings and significance.

## Figures and Tables

**Table 1:** Fasting insulin, HOMA index, and blood glucose in the treatment and control groups at baseline (T0) and after 6 months (T6). Values are expressed as median [25th percentile–75th percentile].

	Treatment Group		Control Group	
	T0	T6	T0	T6
Fasting insulin (mcU/dL)	19.70 [16–26.10]	13.70 [12.10–18.70]	15.65 [14.72–24.37]	14.25 [12.12–16.20]
Homa index	4.90 [3.97–7.20]	3.45 [3–4.80]	4.20 [3.90–6.35]	3.45 [3.10–4.02]
Blood glucose (mg/dL)	107 [103–113.50]	101 [97.25–103.75]	105.50 [103.75–109.25]	100.50 [95.5–104.50]

**Table 2:** Lipid profile in the treatment and control groups, at baseline (T0) and after 6 months (T6). Values are indicated as median [25th percentile–75th percentile]. *** *p* < 0.001 vs. T0; ^##^ *p* < 0.01 vs. Treatment group.

	Treatment Group		Control Group	
	T0	T6	T0	T6
Total cholesterol (mg/dL)	237.50 [229.50–259]	222 [208–241]	244.00 [232.75–274.75]	230.5 [211.75–252]
HDL cholesterol (mg/dL)	39.50 [35.25–43.50]	49.00 [44–51]	41 [37–46]	51 [48–54.25]
LDL cholesterol (mg/dL)	156 [145–168.75]	146 [124–163]	156 [147.25–176.50]	140.50 [123.75–160.50]
Triglycerides (mg/dL)	213.50 [193.25–239]	162 *** [155–172]	226 [208.25–255]	189.50 ***^,##^ [174.50–208.75]

**Table 3:** BMI, weight, and waist circumference in the treatment and control groups at baseline (T0) and after 6 months (T6). Values are indicated as median [25th percentile–75th percentile]. *** *p* < 0.001 vs. T0.

	Treatment Group		Control Group	
	T0	T6	T0	T6
BMI	31.30 [30.50–33.27]	29 *** [28.3–31]	34.25 [32.40–37]	32.75 [30.90–34.52]
Weight (kg)	84.75 [79.25–96.77]	79.20 [75.40–89.90]	90.8 [86.35–100.75]	88.8 [82.37–96.80]
Waist circumference (cm)	98 [96–110]	92 [90.50–105]	103 [95.75–110.50]	100 [90.75–105.50]

## Data Availability

Data are available from the corresponding author on a reasonable request.
